# Special Issue “Microalgal Molecules and Enzymes 2.0”

**DOI:** 10.3390/ijms27052458

**Published:** 2026-03-07

**Authors:** Chiara Lauritano, Assunta Saide

**Affiliations:** Ecosustainable Marine Biotechnology Department, Stazione Zoologica Anton Dohrn, Via A. F. Acton 55, 80133 Napoli, Italy

From lipids to carbohydrates, from pigments to complex polyketides, microalgae have been shown to be possible producers of key high-value molecules with possible applications in various industrial sectors [1,2,3,4,5,6,7,8,9,10]. Advancement in photobioreactor designing and technologies allowing for controlled microalgal culturing favor their growth in the desired conditions and increase the yields of the molecules of interest [11,12,13].

In the current Special Issue, two papers were focused on key microalgal compounds. In particular, Méresse et al. [14] studied the molecule marennine from the cosmopolitan marine pennate diatom *Haslea ostreariai.* Marennine is a blue pigment known for being responsible for the greening of filter-feeding organisms, such as oysters. In addition, it is known to exert antibacterial, antioxidant and antiproliferative activity with potential human health applications. Méresse et al. focused on its effects when tested at 10 and 50 µg/mL on primary cultures of neuroglial cells and showed neuroinflammatory and cell anti-migratory activities. Montuori et al. [15] instead focused on lutein, a carotenoid reported to have antipredator and UV protective effects in the natural environment, as well as multiple possible applications for human health, such as antioxidant, anticancer, anti-inflammatory, and cardioprotective activity. They also discussed recent trends versus green extraction technologies such as the use of green solvents or extraction with ultrasounds, supercritical CO_2_ and microwave-assisted extraction.

Recent approaches for the study of microalgal molecules and enzymes include the use of omics technologies, genetic engineering and system biology. In this Special Issue on “Microalgal Molecules and Enzymes 2.0”, Suwannachuen et al. [16] studied the proteome of *Chlamydomonas* strain CC-4414 exposed to high light stress. Results showed membrane protein variations upon high light exposure with the formation of palmelloid structures and protection from stress through up-regulation of reactive oxygen species management mechanisms and prevention of cell death.

Finally, two papers have focused on genetic engineering methods to produce, respectively, the cannabinoid precursor olivetolic acid (OA) and δ-aminolevulinic acid (ALA), which may have applications in cosmetic, agricultural and pharmaceutical sectors. Awwad and collaborators [17] worked on the diatom species *Phaeodactylum tricornutum*, a model alga with a fast growth rate, for which genome, transcriptome and genetic engineering tools are available [18,19,20,21,22,23]. Their study focused on the introduction of the cannabis genes tetraketide synthase (TKS) and olivetolic acid cyclase (OAC) to produce OA. Western blot analyses confirmed the production of the recombinant TKS and OAC enzymes, while HPLC/UV spectrum detected the OA (0.6–2.6 mg/L).

Regarding the δ-aminolevulinic acid, Kanwal and De-Eknamkul [24] worked with the model cyanobacterium *Synechocystis* sp. PCC 6803. They disrupted the γ-aminobutyric acid (GABA) shunt route by inactivating the gene-encoding glutamate decarboxylase (Gdc). The generated strain produced higher ALA levels with respect to the control. Considering that abiotic stress factors are well known to induce metabolic shifts in microalgae, the authors also used a competitive inhibitor of porphobilinogen synthase, levulinic acid (LA), modified salinity (10 mM NaCl), variation in temperatures (cold exposure at 4 °C) as well as the addition of glucose, succinate, glutamate and glycine. Results showed that the highest ALA concentration was obtained by glucose conditions with glutamate supplementation resulting in about 360 ng g^−1^ cell dry weight of ALA, corresponding to more than 300-fold higher accumulation compared to *Synechocystis* sp. PCC 6803 grown in control conditions.

Altogether, these studies highlight the increasing interest in microalgae for the exploitation of their compounds in different industrial sectors and different methodologies to study and implement the production of the desired products.

## Data Availability

Not applicable.

## References

[1] Miceli M., Cutignano A., Conte M., Ummarino R., Romanelli A., Ruvo M., Leone M., Mercurio F.A., Doti N., Manzo E. (2019). Monoacylglycerides from the Diatom *Skeletonema marinoi* Induce Selective Cell Death in Cancer Cells. Mar. Drugs.

[2] Ávila-Román J., García-Gil S., Rodríguez-Luna A., Motilva V., Talero E. (2021). Anti-Inflammatory and Anticancer Effects of Microalgal Carotenoids. Mar. Drugs.

[3] Riccio G., De Luca D., Lauritano C. (2020). Monogalactosyldiacylglycerol and Sulfolipid Synthesis in Microalgae. Mar. Drugs.

[4] Verma A., Kohli G.S., Harwood D.T., Ralph P.J., Murray S.A. (2019). Transcriptomic Investigation into Polyketide Toxin Synthesis in *Ostreopsis* (Dinophyceae) Species. Environ. Microbiol..

[5] Converti A., Casazza A., Ortiz E., Perego P., Del Borghi A. (2009). Effect of Temperature and Nitrogen Concentration on the Growth and Lipid Content of *Nannochloropsis oculata* and *Chlorella vulgaris* for Biodiesel Production. Chem. Eng. Process. Process Intensif..

[6] Saide A., Martínez K.A., Ianora A., Lauritano C. (2021). Unlocking the Health Potential of Microalgae as Sustainable Sources of Bioactive Compounds. Int. J. Mol. Sci..

[7] Guil-Guerrero J.L., Prates J.A.M. (2025). Microalgae Bioactives for Functional Food Innovation and Health Promotion. Foods.

[8] Ferreira de Oliveira A.P., Bragotto A.P.A. (2022). Microalgae-Based Products: Food and Public Health. Future Foods.

[9] Yarkent Ç., Gürlek C., Oncel S.S. (2020). Potential of Microalgal Compounds in Trending Natural Cosmetics: A Review. Sustain. Chem. Pharm..

[10] Prates J.A.M. (2025). Applications of Bioactive Compounds from Marine Microalgae in Health, Cosmetics, and Functional Foods. Appl. Sci..

[11] Penloglou G., Pavlou A., Kiparissides C. (2024). Recent Advancements in Photo-Bioreactors for Microalgae Cultivation: A Brief Overview. Processes.

[12] He L., Yang W., Guan C., Yan H., Fu P. (2017). Microalgal Cultivation and Hydrodynamic Characterization Using a Novel Tubular Photobioreactor with Helical Blade Rotors. Bioprocess Biosyst. Eng..

[13] Abdur Razzak S., Bahar K., Islam K.M.O., Haniffa A.K., Faruque M.O., Hossain S.M.Z., Hossain M.M. (2024). Microalgae Cultivation in Photobioreactors: Sustainable Solutions for a Greener Future. Green Chem. Eng..

[14] Méresse S., Gateau H., Tirnan T., Larrigaldie V., Casse N., Pasetto P., Mouget J.-L., Mortaud S., Fodil M. (2023). Haslea Ostrearia Pigment Marennine Affects Key Actors of Neuroinflammation and Decreases Cell Migration in Murine Neuroglial Cell Model. Int. J. Mol. Sci..

[15] Montuori E., Lima S., Marchese A., Scargiali F., Lauritano C. (2024). Lutein Production and Extraction from Microalgae: Recent Insights and Bioactive Potential. Int. J. Mol. Sci..

[16] Suwannachuen N., Leetanasaksakul K., Roytrakul S., Phaonakrop N., Thaisakun S., Roongsattham P., Jantasuriyarat C., Sanevas N., Sirikhachornkit A. (2023). Palmelloid Formation and Cell Aggregation Are Essential Mechanisms for High Light Tolerance in a Natural Strain of *Chlamydomonas reinhardtii*. Int. J. Mol. Sci..

[17] Awwad F., Fantino E.I., Héneault M., Diaz-Garza A.M., Merindol N., Custeau A., Gélinas S.-E., Meddeb-Mouelhi F., Li J., Lemay J.-F. (2023). Bioengineering of the Marine Diatom *Phaeodactylum tricornutum* with Cannabis Genes Enables the Production of the Cannabinoid Precursor, Olivetolic Acid. Int. J. Mol. Sci..

[18] Ait-Mohamed O., Novák Vanclová A.M.G., Joli N., Liang Y., Zhao X., Genovesio A., Tirichine L., Bowler C., Dorrell R.G. (2020). PhaeoNet: A Holistic RNAseq-Based Portrait of Transcriptional Coordination in the Model Diatom *Phaeodactylum tricornutum*. Front. Plant Sci..

[19] Hickland P.R., Geisler K., Newsad S., Pasquina M.L., Faessler A.C., Mendoza-Ochoa G.I., Holzer A., Mehrshahi P., Smith A.G. (2025). A New Tool for Engineering *Phaeodactylum tricornutum*: The METE Promoter Drives Both High Expression and B12 -tuneable Regulation of Transgenes. Plant J..

[20] Villar E., Zweig N., Vincens P., Cruz de Carvalho H., Duchene C., Liu S., Monteil R., Dorrell R.G., Fabris M., Vandepoele K. (2025). DiatOmicBase: A Versatile Gene-centered Platform for Mining Functional Omics Data in Diatom Research. Plant J..

[21] Toustou C., Boulogne I., Gonzalez A.-A., Bardor M. (2024). Comparative RNA-Seq of Ten Phaeodactylum Tricornutum Accessions: Unravelling Criteria for Robust Strain Selection from a Bioproduction Point of View. Mar. Drugs.

[22] Bowler C., Allen A.E., Badger J.H., Grimwood J., Jabbari K., Kuo A., Maheswari U., Martens C., Maumus F., Otillar R.P. (2008). The *Phaeodactylum* Genome Reveals the Evolutionary History of Diatom Genomes. Nature.

[23] Nymark M., Sharma A.K., Sparstad T., Bones A.M., Winge P. (2016). A CRISPR/Cas9 System Adapted for Gene Editing in Marine Algae. Sci. Rep..

[24] Kanwal S., De-Eknamkul W. (2023). A Non-Functional γ-Aminobutyric Acid Shunt Pathway in Cyanobacterium *Synechocystis* sp. PCC 6803 Enhances δ-Aminolevulinic Acid Accumulation under Modified Nutrient Conditions. Int. J. Mol. Sci..

